# Are We Progressing?

**Published:** 1871-12

**Authors:** W. E. Driscoll

**Affiliations:** Bedford, Ind.


### ARE WE PROGRESSING?

BY W. E. DRISCOLL.

While reading the report of the proceedings of the Kansas State Dental Society, at its meeting of September, 1871, we were most forcibly impressed by comparing their discussions with those of societies that have been over the same ground several years ago, and are now investigating questions it would seem our Kansas brethren have not as yet heard of.

We would not be understood as desiring to cast any discredit upon them because of not having our advantages, but desire to use the circumstance as an illustration—and it is a most forcible and palpable one—of what a few years of associated effort will do toward improvement in modes of practice. Let some who are disposed to discourage further exertion in this direction compare this report with one of the Lake Erie Dental Association, published in the same number of the DENTAL REGISTER, (November, 1871,) and then affirm, if he can, that the spirit of investigation and improvement that pervades the *better part* of the profession is without tangible—yea, the grandest results.

In support of our view, we might compare numerous passages from each of these reports, but shall refrain, so that every one may do so for himself; and then, if he does not feel that "it (dental knowledge) does move," we can assure him he may very properly indulge in "a little retracing of steps"—just enough to take him out of a profession he can not appreciate.

BEDFORD, IND., December 1, 1871.

